# In Vitro Screening of NaCl-Tolerant Dark Septate Endophytes and Their Growth-Promoting Effects on *Anemone tomentosa*

**DOI:** 10.3390/microorganisms13061303

**Published:** 2025-06-04

**Authors:** Xueyu Jin, Lingjie Xu, Mengyu Dong, Zhanwei Song, Xiaohan Zhang, Wenxiao Liu, Jinge Xu, Yanhui Li

**Affiliations:** 1 College of Landscape Architecture and Tourism, Hebei Agricultural University, Baoding 071000, China; 20232160567@pgs.hebau.edu.cn (X.J.); 20221160074@pgs.hebau.edu.cn (L.X.); songzw417@163.com (Z.S.); h20000411@163.com (X.Z.); 20247161658@pgs.hebau.edu.cn (W.L.); 20242160587@pgs.hebau.edu.cn (J.X.); 2 Shijiazhuang Landscaping Management and Maintenance Center, Shijiazhuang 050000, China; dmengyu1@163.com; 3 Hebei Key Laboratory of Floral Biological Breeding, Baoding 071000, China; 4 National Engineering and Technology Center for Northern Mountain Agriculture, Baoding 071000, China

**Keywords:** dark septate endophytes, NaCl stress, strain screening, antioxidant defense response, fungal inoculation

## Abstract

NaCl is the main cause of natural soil salinization. Exploring dark septate endophytes (DSEs) with NaCl tolerance provides information for ecological remediation in saline soil areas. In this study, six DSE strains (*Didymella macrostoma (Dm)*, *Paraboeremia selaginellae* (*Ps*), *Paraphoma pye* (*Pp*), *Paraphoma aquatica* (*Pa*), *Acrocalymma ampeli* (*Aa*), and *Exophiala xenobiotica* (*Ex*)) isolated from the root sections of *Anemone tomentosa* were subjected to in vitro NaCl stress experiments and inoculation tests. The results showed that six DSE strains can grow on solid media with different NaCl concentrations (0, 0.2, 0.4, 0.6, 0.8, and 1.0 M) and increase the antioxidant enzyme activities and soluble protein contents to adapt to a salt stress environment. Among these strains, the *Pp* strain exhibited the greatest biomass accumulation under high NaCl concentrations (1.0 M), indicating greater NaCl tolerance compared to the other five strains. In addition, in the pot experiment, all six DSE strains were able to successfully establish a symbiotic relationship with *A. tomentosa*, and the *Pp* strain also showed significant growth-promoting effects on seedlings. In summary, the *Pp* strain is identified as having strong NaCl tolerance and a significant growth-promoting impact, indicating that it has potential applications as a NaCl-tolerant microbial agent and can be used for bioremediation in saline soils. This research contributes to the basic material and theoretical basis for joint plant–microbe combined remediation in areas prone to soil salinization.

## 1. Introduction

Soil salinization has become one of the major problems threatening the global biosphere and the ecological environment [1]. Excessive soil salinity can be detrimental to plant growth, leading to disruptions in osmoregulation and redox homeostasis in plants, which in turn impair plant metabolism and ultimately lead to plant death [2,3,4]. NaCl is the primary contributor to soil salinization. Excess Na^+^ and Cl^−^ accumulation causes oxidative damage and ionic imbalances in plant tissues, negatively affecting plant metabolism [5]. Microbe-assisted phytoremediation is considered an effective strategy for enhancing plant resilience to salt stress [6]. Symbiotic fungi have been reported to affect the growth characteristics of their host plants or induce the production of chemical substances, such as proteins, polysaccharides, and hormones, which increase plant growth under saline conditions [7,8]. Therefore, the introduction of beneficial microorganisms can improve the salt tolerance of plants for normal growth in saline soils.

Dark septate endophytes (DSEs) are a group of endophytic fungi characterized by dark septal hyphae and microsclerotia. They inhabit a variety of root tissues, including epidermal cells, the root cortex, vascular tissues, and intercellular spaces [9]. DSEs are widely distributed across natural ecosystems and are particularly prevalent in extreme environments, such as saline soils, arid regions, and polluted habitats [10,11,12,13]. They form symbiotic relationships with a wide range of plants; the host plants of DSEs include nearly 600 species of 114 families and 320 genera [14]. DSEs play an important role in ecological restoration, contributing to plant growth promotion and thereby improving plant resistance under stressful conditions [15,16,17]. Qu et al. [18] reported that inoculation with DSEs enhanced the growth of blueberry seedlings and boosted antioxidant activity in their root systems under salt stress conditions. Tan et al. [19] reported that inoculation with DSEs significantly promotes root system development and enhances biomass accumulation in *Glycyrrhiza glabra* L. (Fabaceae) under salt stress. Similarly, Hou et al. [20] reported that under NaCl stress, inoculation with DSEs can increase indole-3-acetic acid (IAA) levels while reducing sodium (Na⁺) concentrations in the root system of *Artemisia ordosica* Krasch. (Asteraceae). In summary, these findings suggest that DSEs alleviate the detrimental effects of NaCl stress in host plants by promoting biomass production, enhancing antioxidant enzyme activities and endogenous growth hormone levels, and maintaining Na⁺/K⁺ homeostasis, thereby improving overall plant resilience under salt stress. Furthermore, DSEs promote the absorption and transformation of mineral nutrients [21,22,23]. Currently, studies on DSEs have focused primarily on their resource distribution, isolation, identification, and impact on host plants, with limited research on their stress resistance mechanism. Owing to the low host specificity of DSEs [24], selected DSE strains can also be applied to nonhost plants [15]. As a result, targeted screening of DSE strains under controlled experimental conditions (drought, heavy metal, and saline–alkali stresses) has become a current research focus [17].

*Anemone tomentosa* (Maxim.) C.Pei (Ranunculaceae), a perennial plant in China, is distributed in the northwest and southwest of China [25]. This wildflower is valued for its high ornamental and medicinal properties. The root of *A. tomentosa* is rich in various chemical components, including flavonoids, saponins, and polysaccharides, which exhibit antitumor and anti-inflammatory effects [26,27]. It can also thrive even in complex environmental conditions because of its strong resistance and adaptability to minimal management [28,29]. Microscopic staining of the root system revealed a high abundance of symbiotic fungi [30], providing a strong theoretical foundation for the future development and application of endophytic fungi associated with *A. tomentosa*. Therefore, in this study, two experiments were conducted with six DSE strains isolated from the roots of *A. tomentosa*: (1) the response of six DSE strains under different NaCl stresses in pure culture conditions, and (2) effects of inoculation *of A. tomentosa* with these DSE strains on its growth and nutrient uptake in a pot experiment. Through a combination of in vitro experiments and inoculation tests, we aimed to explore the influence of DSEs on the growth of *A. tomentosa*. The findings of this study contribute to understanding the symbiotic mechanism between DSEs and *A. tomentosa*, facilitate the construction of an excellent plant–endophytic fungi interaction system, and provide material support and a theoretical basis for the restoration of the saline ecological environments.

## 2. Materials and Methods

### 2.1. Biological Materials

Six DSE species were isolated from root samples of *A. tomentosa* and were identified based on morphological characteristics (Figure S1) and phylogenetic analysis of nuclear ribosomal DNA (nrDNA) internal transcribed spacer (ITS) sequences (Figure S2).

Roots were washed under sterile water and cut into 2.0–2.5 cm segments with a sterilized scalpel. The root segments were surface-sterilized sequentially with 75% ethanol followed by 10% sodium hypochlorite, then rinsed multiple times with sterile water and dried on sterile filter paper. Then, these root segments were cultured on potato dextrose agar (PDA; selected from Solarbio Science & Technology Co., Ltd., Beijing, China) and incubated in the dark at 28 °C and observed once a day. When dark mycelial gbrowth first appeared, actively growing hyphae were inoculated into a new PDA medium for purification and incubated in the dark at 28 °C. At the colony edge of the purified strain, 1/3 of the coverslip was inserted into the medium at 45° and incubated in the dark at a constant temperature of 28 °C. The morphological characteristics of the colonies were observed and recorded at 2d intervals. The coverslips with mycelia were removed and observed under a light microscope, and the mycelia color, morphology, and spore morphology were recorded for morphological identification.

Fresh mycelia (20 mg) were scraped and ground thoroughly, and mycelial DNA was extracted using a fungal genomic DNA extraction kit (provided by Beijing Solarbio Science & Technology Co., Ltd., Beijing, China) and sequenced. The complete ITS sequences of the measured DSE strains were submitted to GenBank for BLAST (https://blast.ncbi.nlm.nih.gov/Blast.cgi) comparison, and the phylogenetic tree was constructed using MEGA version 6.0 software. A combination of morphological features and molecular sequencing was used to determine the taxonomic status of strains. The identified strains included *Didymella macrostoma* (Mont.) Qian Chen and L.Cai (Ascomycota, Didymellaceae); *Paraboeremia selaginellae* (Sacc.) Qian Chen and L.Cai (Ascomycota, Didymellaceae); *Paraphoma pye* Moslemi and P.W.J. Taylor (Ascomycota, Phaeosphaeriaceae); *Paraphoma aquatica* Magaña-Dueñas, Stchigel, and Cano-Lira (Ascomycota, Phaeosphaeriaceae); *Acrocalymma ampeli* Tennakoon, C.H.Kuo, and K.D. Hyde (Ascomycota, Acrocalymmaceae); and *Exophiala xenobiotica* de Hoog, J.S. Zeng, Harrak, and Deanna A. Sutton (Ascomycota, Herpotrichiellaceae). These strains were cultured on PDA and maintained at 28 °C in a mold incubator.

Mature seeds of *A. tomentosa* were collected from the Teaching Experimental Base of Hebei Agricultural University and stored at 4 °C. Before germination, surface fluff was removed from the seeds, which were then placed in Petri dishes and incubated at 25 °C under light conditions. Thirty-five seeds were placed per dish, with three replicates established, resulting in a total of 105 seeds. From these, 35 uniformly growing seedlings were selected for the backcrossing experiment prior to inoculation treatment. Germinated seedlings were planted into sterile plastic pots (14 cm × 12 cm × 10 cm) at a rate of one plant per pot, each pot containing 1000 g of sterilized substrate. The substrate was a blend of charcoal soil, garden soil, and river sand in a ratio of 2:1:1. These substrates were passed through a 2 mm sieve and mixed, and the mixture was subsequently autoclaved at 121 °C for a duration of 2 h. After 3 months of planting, 35 uniformly growing seedlings were selected for the inoculation test. During seedling cultivation, plants were moderately irrigated with sterile water to support normal growth.

### 2.2. Experimental Design

#### 2.2.1. In Vitro NaCl Stress Test

NaCl was used to simulate salt stress. The experiment was carried out on modified Melin Norkrans (MMN; selected from Shanghai Ruichu Biotech Co., Ltd., Shanghai, China) media under sterile conditions. Six different DSE strains were used, namely *Didymella macrostoma* (*Dm*), *Paraboeremia selaginellae* (*Ps*), *Paraphoma pye* (*Pp*), *Paraphoma aquatica* (*Pa*), *Acrocalymma ampeli* (*Aa*), and *Exophiala xenobiotica* (*Ex*), with six different NaCl stresses: 0 M, 0.2 M, 0.4 M, 0.6 M, 0.8 M, and 1.0 M. Five replicates were used for each treatment with a total of 180 samples. After the DSE colonies were revitalized on PDA media at 28 °C for 15 days, 9 mm fungal disks were inoculated into the MMN media. The solid medium with different NaCl concentrations was then incubated in dark inversion at 28 °C for 15 days. The liquid culture was grown at the same temperature in a thermostatic shaker incubator at 180 rpm for 15 days in the dark. After 15 days of incubation, the mycelia were harvested for relevant index measurements.

#### 2.2.2. Pot Experiment

The pot experiment was conducted under natural light conditions at Hebei Agricultural University. Seven inoculation treatments were established: single inoculations with *Dm*, *Ps*, *Pp*, *Pa*, *Aa*, and *Ex*, and a non-inoculated control (CK). Five replicates were set up for each treatment, with a total of 35 pots.

For the preparation of fungal inoculum, 9 mm mycelial disks were cultured in 150 mL of MMN for 15 d at 28 °C, 180 rpm/min continuous shaking on a thermostatic incubator. The mycelia were then homogenized with sterile water (*v*:*v* = 3:1) and inoculated into the seedlings through perforated root irrigation. Each pot was inoculated with 20 mL of mycelial suspension, whereas the control group was inoculated with 20 mL of sterile MMN liquid media. Three months after the seedlings were planted, 35 uniformly growing seedlings were selected for the inoculation test, and after 15 days, the seedlings were treated with another inoculation treatment, with a total of two inoculation treatments. After 60 days of DSE inoculation, the seedlings were harvested.

### 2.3. Determination of DSE Growth Indicators

At the end of the culture period, the cross-measurement technique was used to determine the colony diameter, and pictures were taken to record the morphology of the colonies. At the end of the liquid culture, the fungal mycelia were filtered and then weighed after drying at 80 °C to determine the biomass production.

### 2.4. Determination of DSE Physiological Indicators

Before the DSE strains were cultured, the pH of the culture solution was determined to be 5.5, and after the liquid culture, the fungal mycelia were filtered, and a pH meter was used to measure and record the pH of the culture solution.

The nitroblue tetrazolium photoreduction method was used to determine the activity of superoxide dismutase (SOD) [31]. The colorimetric method of Thomas Brilliant Blue was used to determine the soluble protein content [32]. The thiobarbituric acid method was used to determine the malondialdehyde (MDA) content [33,34,35], while the NaOH extraction method was used to determine the melanin concentration [36]. The anthrone colorimetric method was used to determine the soluble sugar content [37].

### 2.5. Determination of Plant Morphological Indicators

After 60 days of DSE inoculation treatments, a Vernier caliper was used to measure the height and crown breadth of the plants. Then, the shoots and the roots were harvested separately.

### 2.6. Determination of Fungal Colonization and Fungal Growth Response

The fresh roots of *A. tomentosa* were randomly sampled to determine DSE colonization by using the methods of Phillips and Hayman [38].

The fungal growth response was calculated according to the method of Yong Zhou and Lingjie Xu [39,40].

### 2.7. Determination of Plant Nutrient Element

Dried powdery samples (0.2 g) of shoots and roots were digested using the H_2_SO_4_-H_2_O_2_ method, and the digestion solution was fixed with deionized water to 50 mL. The nitrogen (N) content was measured by the Kjeldahl method, the phosphorus (P) content was measured by the molybdenum antimony colorimetric method, and the potassium (K) content was measured by the flame atomic absorption spectrometer [41].

### 2.8. Determination of Photosynthetic Pigment Contents

The ethanol extraction method [42] was used to determine the photosynthetic pigment (carotenoid and chlorophyll) contents.

### 2.9. Statistical Analysis

For the in vitro experiments, a two-way analysis of variance (ANOVA) was performed to evaluate the effects of NaCl concentration and fungal species on the growth and physiological responses of the six DSE strains. For the inoculation experiment, a one-way ANOVA was employed to assess the impact of fungal inoculation on the growth and physiological traits of *A. tomentosa*. Statistical analyses were conducted using SPSS 25.0, with significant differences determined by Duncan’s multiple-range test at a significance level of *p* < 0.05. Data processing was carried out in Microsoft Excel, and statistical charts, principal component analysis (PCA), and correlation heatmaps were generated using Origin Version 2022. Hierarchical clustering analysis and variation partitioning analysis (VPA) were conducted with the “pheatmap” and “vegan” packages in “R version 4.4.2”.

## 3. Results

### 3.1. Effects of Different NaCl Concentrations on the Morphology of DSE Strains

**Figure 1:**
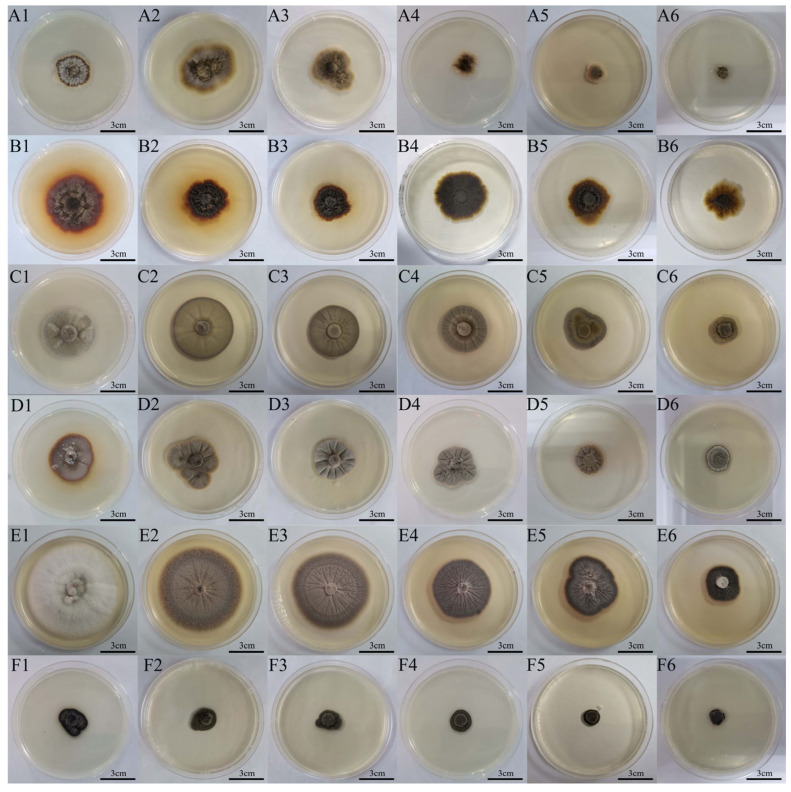
The colony morphology of six dark septate endophytes (DSEs) in Melin Norkrans (MMN) medium under different NaCl concentrations. (**A1**–**A6**) Colony morphology of *Didymella macrostoma* (*Dm*) under 0, 0.2, 0.4, 0.6, 0.8, and 1.0 M NaCl stress, respectively. (**B1**–**B6**) Colony morphology of *Paraboeremia selaginellae* (*Ps*) under 0, 0.2, 0.4, 0.6, 0.8, and 1.0 M NaCl stress, respectively. (**C1**–**C6**) Colony morphology of *Paraphoma pye* (*Pp*) under 0, 0.2, 0.4, 0.6, 0.8, and 1.0 M NaCl stress, respectively. (**D1**–**D6**) Colony morphology of *Paraphoma aquatica* (*Pa*) under 0, 0.2, 0.4, 0.6, 0.8, and 1.0 M NaCl stress, respectively. (**E1**–**E6**) Colony morphology of *Acrocalymma ampeli* (*Aa*) under 0, 0.2, 0.4, 0.6, 0.8, and 1.0 M NaCl stress, respectively. (**F1**–**F6**) Colony morphology of *Exophiala xenobiotica* (*Ex*) under 0, 0.2, 0.4, 0.6, 0.8, and 1.0 M NaCl stress, respectively. Scale bars = 3 cm.

The morphological changes in the colonies of six DSE strains under different NaCl concentrations are shown in Figure 1. As NaCl stress increased, the colony color of the *Dm* strain gradually became lighter. At 0 M, the color of the *Ps* colony was brown-yellow in the middle and orange-yellow around, with dense mycelia. The colony color of *Ps* gradually became darkened with the increasing NaCl stress. Similarly, the *Pa* colony transitioned from brownish yellow to light gray, and radial grooves appeared on the colony surface. At 0 M, the *Pp* strain showed radial grooves on the surface, and the colony color changed to grayish black with increasing stress. At 0 M, the colony color of *Aa* was white with loose mycelia, the color became darker with increasing stress, and the surface of the colony presented radial grooves. The *Ex* strain produced black colonies with aerial mycelia in the absence of NaCl stress, but the aerial mycelium decreased with increasing stress.

### 3.2. Effects of Different NaCl Concentrations on Growth Indicators of DSE Strains

**Figure 2:**
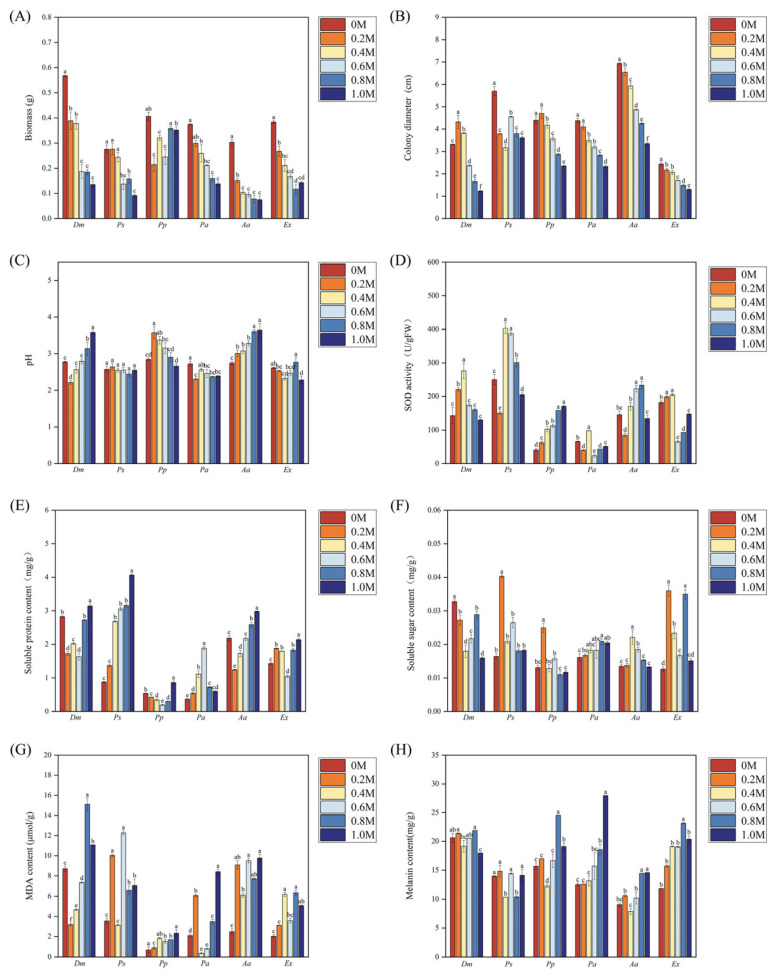
Effects of different NaCl stress on the biomass (**A**), colony diameter (**B**), pH (**C**), superoxide dismutase (SOD) activity (**D**), soluble protein content (**E**), soluble sugar content (**F**), malondialdehyde (MDA) content (**G**), and melanin content (**H**) of *Didymella macrostoma* (*Dm*), *Paraboeremia selaginellae* (*Ps*), *Paraphoma pye* (*Pp*), *Paraphoma aquatica* (*Pa*), *Acrocalymma ampeli* (*Aa*), and *Exophiala xenobiotica* (*Ex*). The concentrations of NaCl stress are indicated as 0, 0.2, 0.4, 0.6, 0.8, and 1.0 M. The error bars represent the mean ± standard error (SE) of three independent biological replicates (*n* = 3). Different letters above the error bars indicate a significant difference at *p* < 0.05 by Duncan’s multiple-range test.

The two-way ANOVA results (Table 1) indicated that both DSE species and NaCl stress had significant effects on the diameter, biomass, pH, soluble protein content, SOD activity, melanin content, MDA content, and soluble sugar content of six DSE strains. All six DSE strains were able to grow under different NaCl stresses. As the level of NaCl stress increased, the biomass and colony diameter of the *Ps*, *Pa*, *Ex*, and *Aa* strains generally declined (Figure 2A,B). The *Dm* and *Pp* strains reach their maximum values at 0.2 M NaCl. Under high NaCl stress (1.0 M), the biomass of the *Pp* strain was significantly greater than that of the other five strains, indicating its superior NaCl tolerance.

### 3.3. Effects of Different NaCl Concentrations on Physiological Indicators of DSE Strains

#### 3.3.1. pH of the DSE Culture Solution

Under different concentrations of NaCl, six DSE strains showed significant differences in culture solution pH (Figure 2C). The pH of the culture solution for the *Dm* and *Ex* strains initially decreased and then increased, whereas that of the *Pp* strain showed the opposite trend, first increasing and then decreasing, reaching a minimum value of 3.87 at 1.0 M. Except at 0.4 M, the culture solution pH of the *Pa* was significantly lower than the control, with reductions of 15.3%, 9.7%, 13.0%, and 12.3%, respectively. In contrast to the other five strains, the pH of the culture solution of the *Ps* strain did not significantly differ across different treatments.

#### 3.3.2. SOD Activity of DSE Strains

As NaCl stress increased, the SOD activity of the *Dm*, *Ps*, and *Aa* strains exhibited an initial rise followed by a decline (Figure 2D). The *Aa* strain reached its maximum value of 233.24 U/g at 0.8 M NaCl, while the *Dm*, *Ps*, *Pa*, and *Ex* strains achieved their highest SOD activity at 0.4 M NaCl, with significant increases of 93.2%, 61.3%, 49.0%, and 12.4%, respectively. In contrast, the SOD activity of the *Pp* strain continuously increased with rising NaCl concentrations. Under high NaCl stress, the SOD activity of *Pp* was found to be significantly higher than the control, indicating that the *Pp* strain was superior to the other five DSE strains in enhancing antioxidant enzyme activity in response to NaCl stress.

#### 3.3.3. Soluble Substance Content of DSE Strains

The soluble protein of the *Pa* strain initially increased and then declined with rising NaCl stress, reaching its maximum at 0.6 M NaCl (Figure 2E). The *Ps* and *Ex* strains showed a continuous increase in the soluble protein content with increasing NaCl concentrations, whereas the *Dm*, *Pp*, and *Aa* strains exhibited an initial decrease followed by an increase with increasing NaCl concentrations. At 1.0 M, *Dm*, *Pp*, and *Aa* strains reached their maximum soluble protein contents, with increases of 11%, 59%, and 36%, respectively, compared to the control. Overall, among these six DSE strains, the *Ps* strain showed superior soluble protein accumulation across all NaCl concentrations.

The soluble sugar contents of *Ps*, *Pp*, *Aa*, and *Ex* first increased and then decreased with increasing NaCl stress, in which *Ps*, *Pp*, and *Ex* reached their maximum values at 0.2 M NaCl (Figure 2F), increasing by 11%, 59%, and 36%, respectively, compared to the control. The *Aa* strain reaches its maximum value of 0.022 mg/g at 0.4 M NaCl. In contrast, the soluble sugar content of *Dm* decreased with increasing stress, being significantly lower in all the treatments than in the control.

#### 3.3.4. Melanin and MDA Content of DSE Strains

The six DSE strains exhibited distinct patterns in MDA accumulation under different NaCl stress (Figure 2G). For *Pp* and *Aa* strains, the MDA content showed an overall increase with increasing NaCl concentration. The *Ps* and *Ex* strains showed a rise followed by a decline. However, the MDA contents of the *Dm* and *Pa* strains first decreased and then increased. Under medium NaCl stress (0.4 M and 0.6 M), the MDA content in the *Dm* and *Pa* strains was significantly reduced compared to the control, suggesting that these two DSE strains possess more effective mechanisms for mitigating oxidative stress than the other four DSE strains.

The melanin content of the *Pa* and *Aa* strains displayed an overall increasing trend as salinity increased (Figure 2H). The *Aa* strain consistently maintained higher melanin levels than the control, except at 0.4 M. Meanwhile, the *Ps* strain showed lower melanin content at 0.4 M and 0.8 M, with no notable changes under other concentrations. With increasing stress, the melanin contents of *Ex* and *Pp* first increased before declining, and both reached their maximum values at 0.8 M, with substantial increases of 56.4% and 95.2%, respectively, relative to the control. The *Dm* strain showed a significant decrease in melanin at 1.0 M, though no significant differences were observed at other concentrations.

#### 3.3.5. Principal Component Analysis and Variation Partitioning Analysis

**Figure 3:**
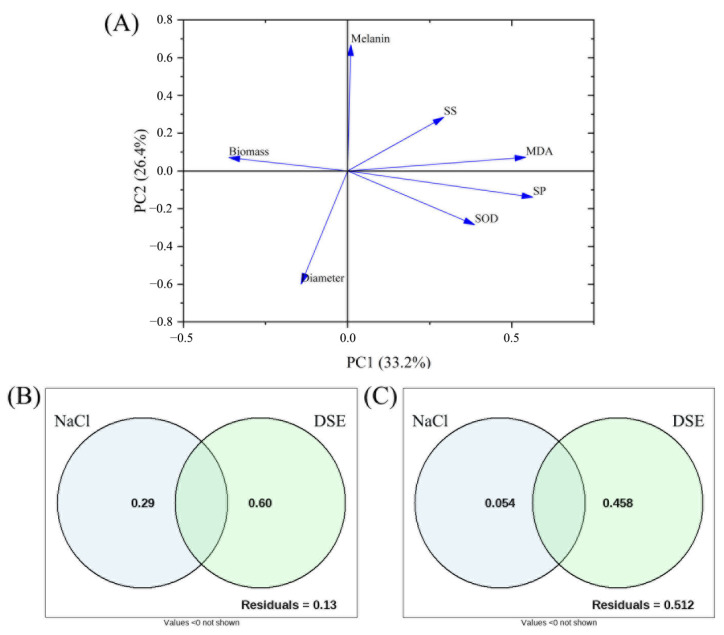
Principal component analysis (PCA) and variance partitioning analysis (VPA) on colony diameter (diameter), biomass, pH, soluble protein (SP) content, superoxide dismutase (SOD) activity, melanin content, malondialdehyde (MDA) content, and soluble sugar (SS) content in the in vitro NaCl stress test. (**A**) PCA plot of growth indicators (diameter and biomass) and physiological indicators (SP, SOD, melanin, MDA, and SP) in the in vitro NaCl stress test. (**B**) VPA plot of the relative effect of NaCl stress (NaCl) and dark septate endophyte (DSE) species on the growth indicators. (**C**) VPA plot of the relative effect of NaCl and DSE species on the physical indicators. Values < 0 not shown.

The PCA results (Figure 3A) show that the first principal component (PC1) and second principal component (PC2) together explain 59.6%. The contributions of PC1 and PC2 were 33.2% and 26.4%, respectively, while MDA, soluble protein, and biomass were the most significant contributing factors.

The VPA results (Figure 3B,C) that DSE species explained 58% and 60% of the growth indicators and physiological indicators, respectively, demonstrated that DSE species are the main factor affecting the NaCl tolerance effect of different strains.

#### 3.3.6. Relationship Between Growth and Physiological Indicators of DSE Strains

**Figure 4:**
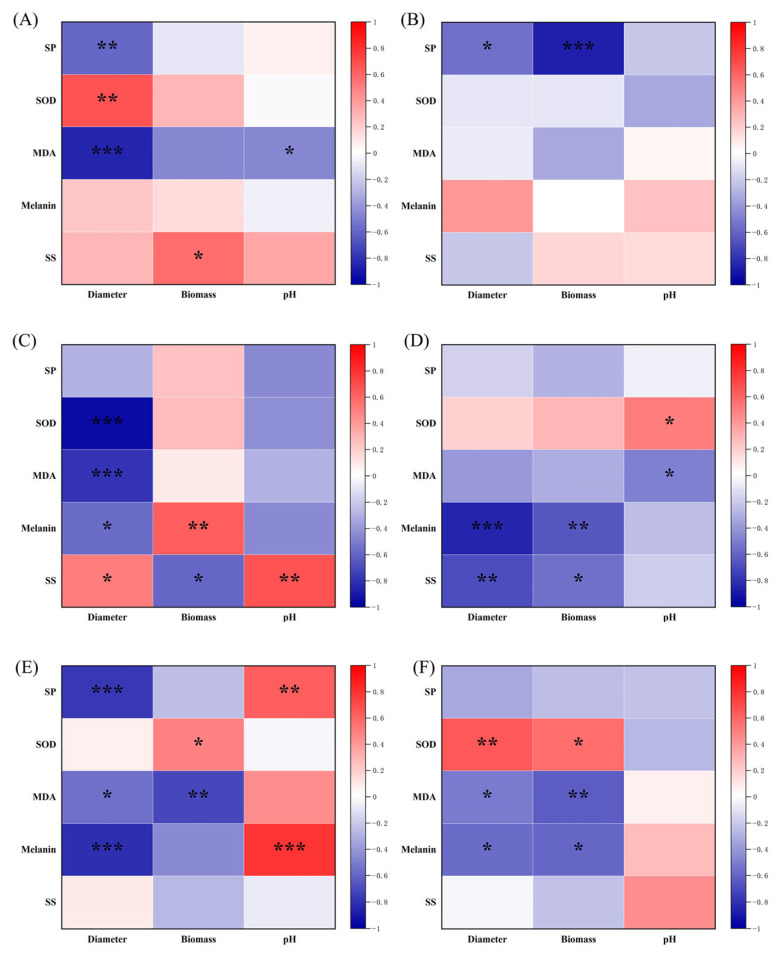
Relationship between diameter, biomass, pH, soluble protein (SP), superoxide dismutase (SOD) activity, melanin, malondialdehyde (MDA), and soluble sugar (SS) content in the in vitro NaCl stress test. (**A**–**F**) *Didymella macrostoma* (*Dm*), *Paraboeremia selaginellae* (*Ps*), *Paraphoma pye* (*Pp*), *Paraphoma aquatica* (*Pa*), *Acrocalymma ampeli* (*Aa*), and *Exophiala xenobiotica* (*Ex*) strains, respectively. The color bar on the right side represents significant R-values, respectively. The various symbols above the bars indicate significant differences between growth indicators (diameter and biomass) and physiological (SP, SOD, melanin, MDA, and SS) indicators (* *p* < 0.05, ** *p* < 0.01, *** *p* < 0.001).

To further understand how NaCl stress influences DSE performance, the relationships among biomass, colony diameter, SOD activity, MDA content, soluble protein, soluble sugar, pH, and melanin were analyzed (Figure 4). Each DSE strain showed distinct correlation patterns. In the *Dm* strain (Figure 4A), biomass was positively related to soluble sugar content, and culture solution pH was negatively associated with MDA content. For *Ps* (Figure 4B), soluble protein content showed negative correlations with both colony diameter and biomass. The *Pp* strain (Figure 4C) displayed a positive correlation between colony diameter and soluble sugar content, while negative correlations were found with MDA, SOD, and melanin. Biomass was positively linked to melanin but negatively to sugar levels. In the *Pa* strain (Figure 4D), both colony diameter and biomass were negatively correlated with melanin and soluble sugar content. However, its pH showed a positive correlation with SOD. In the *Aa* strain (Figure 4E), colony diameter was negatively associated with soluble protein, MDA, and melanin contents. For the *Ex* strain (Figure 4F), both colony diameter and biomass had positive correlations with SOD activity and negative correlations with MDA and melanin contents.

### 3.4. Effects of DSE Inoculation on the Growth Promotion of Anemone tomentosa

#### 3.4.1. Effects of DSE Inoculation on the Growth Indicators of *Anemone tomentosa*

**Figure 5:**
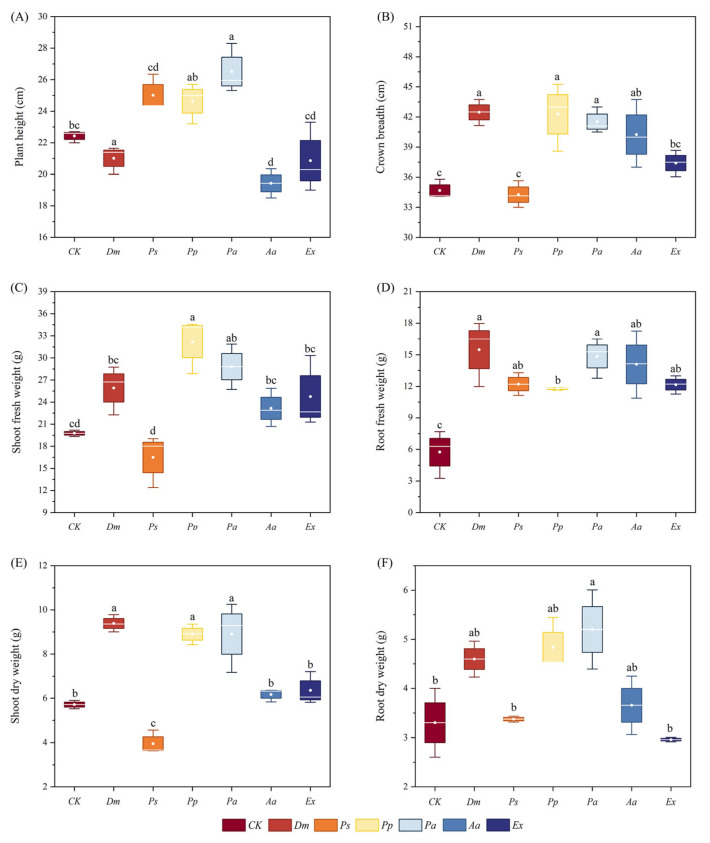
Effects of different inoculation treatments of *Anemone tomentosa* on plant height (**A**), crown breadth (**B**), shoot fresh weight (**C**), root fresh weight (**D**), shoot dry weight (**E**), and root dry weight (**F**). CK indicates the control treatment, *Dm* indicates inoculation of *Didymella macrostoma*, *Ps* indicates inoculation of *Paraboeremia selaginellae*, *Pp* indicates inoculation of *Paraphoma pye*, *Pa* indicates inoculation of *Paraphoma aquatica*, *Aa* indicates inoculation of *Acrocalymma ampeli*, and *Ex* indicates inoculation of *Exophiala xenobiotica*. The error bars represent the mean ± standard error (SE) of three independent biological replicates (*n* = 3). Different letters above the error bars indicate a significant difference at *p* < 0.05 by Duncan’s multiple-range test.

The effects of DSE strains on plants varied (Figure 5). The *Dm* and *Pa* inoculation significantly increased both plant height and crown breadth. In contrast, inoculation with *Ps*, *Aa*, and *Ex* showed a negative effect on plant height. Except for *Ps* and *Ex*, the inoculation of other DSE strains significantly increased the crown breadth of plants, with increases of 22.4%, 21.9%, 19.8%, and 16.1%, respectively, compared to the control. Inoculation with all six DSE strains significantly increased root fresh weight. Inoculation with *Pp* and *Aa* significantly enhanced the root dry weight by 46.5% and 57.5%, respectively. In addition, inoculation with *Dm*, *Pp*, *Pa*, *Aa*, and *Ex* increased shoot fresh weight and dry weight. Overall, inoculation with these five strains had a positive effect on the growth of *A. tomentosa*.

#### 3.4.2. DSE Colonization and Mycorrhizal Growth Response of *Anemone tomentosa*

**Figure 6:**
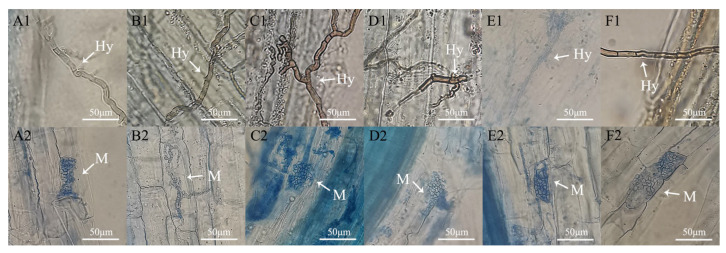
DSE colonization structure in the roots of *Anemone tomentosa*. (**A**–**F**) *Didymella macrostoma* (*Dm*), *Paraboeremia selaginellae* (*Ps*), *Paraphoma pye* (*Pp*), *Paraphoma aquatica* (*Pa*), *Acrocalymma ampeli* (*Aa*), and *Exophiala xenobiotica* (*Ex*). Hy represents the DSE hyphae; M represents the DSE microsclerotia. Scale bars = 50 μm.

**Figure 7:**
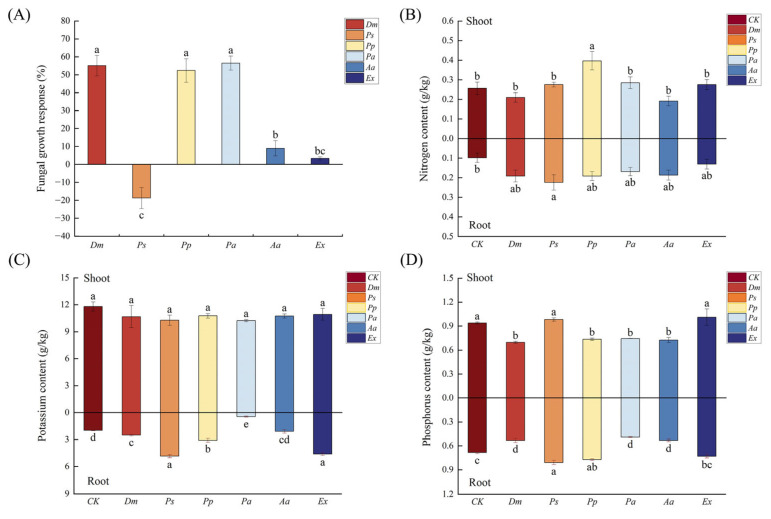
Effects of different inoculation treatments of *Anemone tomentosa* on fungal growth response (**A**), nitrogen content (**B**), potassium content (**C**), and phosphorus content (**D**). CK indicates the control treatment, *Dm* indicates inoculation of *Didymella macrostoma*, *Ps* indicates inoculation of *Paraboeremia selaginellae*, *Pp* indicates inoculation of *Paraphoma pye*, *Pa* indicates inoculation of *Paraphoma aquatica*, *Aa* indicates inoculation of *Acrocalymma ampeli*, and *Ex* indicates inoculation of *Exophiala xenobiotica*. The error bars represent the mean ± standard error (SE) of three independent biological replicates (*n* = 3). Different letters above the error bars indicate a significant difference at *p* < 0.05 by Duncan’s multiple-range test.

Typical DSE structures, including dark septate hyphae and microsclerotia, were observed in the roots of *A. tomentosa* (Figure 6), confirming successful colonization. The fungal growth response of *Dm*, *Pp*, and *Pa* was significantly higher than that of the other three strains (Figure 7A), indicating that these DSE strains had a notably positive impact on the growth of *A. tomentosa*. However, the fungal growth response of *Ps* was negative, indicating that the inoculation with *Ps* had an inhibitory effect on the growth of *A. tomentosa*. Overall, inoculation with *Dm*, *Pp*, and *Pa* promoted seedling growth better than inoculation with *Ps*, *Aa*, and *Ex.*

#### 3.4.3. Effects of DSE Inoculation on Nutrient Elements of *Anemone tomentosa*

Inoculation with all six DSE strains significantly influenced N content in the roots of *A. tomentosa*, with *Ps* having the most significant effect on the N content in the shoot (Figure 7B). *Pp* inoculation had the highest shoot N content with a significant increase of 54.5%. In contrast, the inoculation treatments had minimal impact on K content in the roots, with no significant differences detected among treatments (Figure 7C). Inoculation with *Ps*, *Dm*, *Pp*, and *Ex* significantly enhanced the K content of the root, with the *Ps* inoculation treatment having the most significant effect. Inoculation with *Ps* and *Ex* increased the P content in both shoots and roots of *A. tomentosa* (Figure 7D). In roots, *Ps* inoculation had a more significant effect on the P content, leading to a significant increase of 14.8%.

#### 3.4.4. Effect of DSE Inoculation on Photosynthetic Pigments of *Anemone tomentosa*

**Figure 8:**
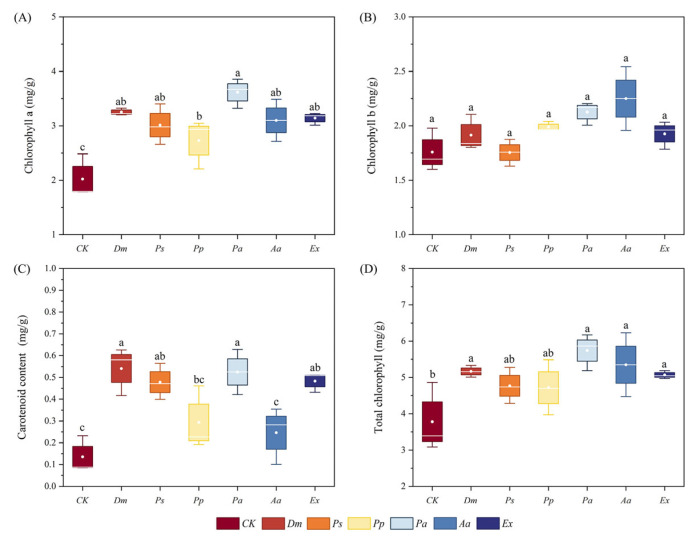
Effects of different inoculation treatments of *Anemone tomentosa* on the chlorophyll a (**A**), chlorophyll b (**B**), carotenoid content (**C**), and total chlorophyll (**D**). CK indicates the control treatment, *Dm* indicates inoculation of *Didymella macrostoma*, *Ps* indicates inoculation of *Paraboeremia selaginellae*, *Pp* indicates inoculation of *Paraphoma pye*, *Pa* indicates inoculation of *Paraphoma aquatica*, *Aa* indicates inoculation of *Acrocalymma ampeli*, and *Ex* indicates inoculation of *Exophiala xenobiotica*. The error bars represent the mean ± standard error (SE) of three independent biological replicates (*n* = 3). Different letters above the error bars indicate a significant difference at *p* < 0.05 by Duncan’s multiple-range test.

Compared with the control, inoculation with all six DSE strains significantly increased the content of chlorophyll a (Figure 8A), with *Pa* showing a maximum value of 3.62 mg/g, a significant increase of 78.8% than the control. For chlorophyll b (Figure 8B), all six DSE inoculation treatments increased chlorophyll b, but no significant differences were detected between these treatments. Except for *Pp* and *Aa*, inoculation with the other DSE strains significantly increased the carotenoid content by 299.5%, 253.3%, 287.6%, and 256.8%, respectively (Figure 8C). Similarly, excluding *Ps* and *Pp*, inoculation with other DSE strains significantly increased the total chlorophyll content (Figure 8D), with the maximum total chlorophyll content found in the *Pa* inoculation treatment with 5.74 μg/g. These findings indicated that inoculation with all six DSE strains increased the content of photosynthetic pigments in *A. tomentosa*, with *Pa* having the most pronounced effect on the synthesis of photosynthetic pigments.

#### 3.4.5. Heatmap Analysis of Correlation Clustering

**Figure 9:**
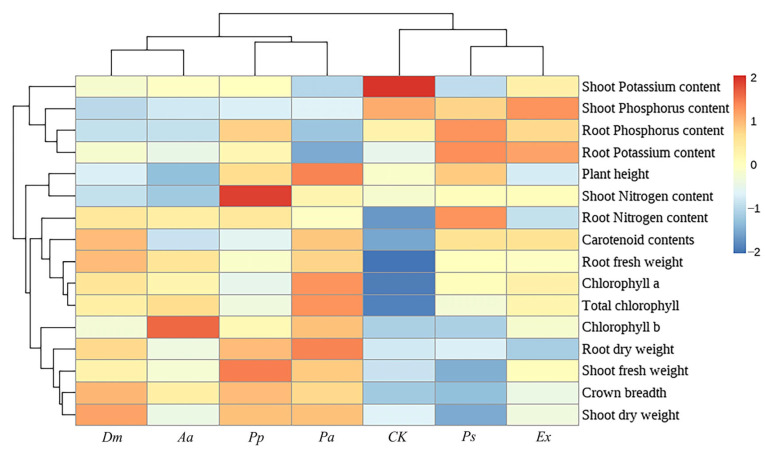
Heatmap showing the hierarchical clustering among the treatments. Analysis was performed by using normalized data of all the parameters. CK indicates the control treatment, *Dm* indicates inoculation of *Didymella macrostoma*, *Ps* indicates inoculation of *Paraboeremia selaginellae*, *Pp* indicates inoculation of *Paraphoma pye*, *Pa* indicates inoculation of *Paraphoma aquatica*, *Aa* indicates inoculation of *Acrocalymma ampeli*, and *Ex* indicates inoculation of *Exophiala xenobiotica*.

The clustered heatmap analysis revealed two distinct treatment groups: inoculations with *Aa*, *Pp*, *Dm*, and *Pa* formed one cluster, while *Ps*, *Ex*, and the control treatment formed the other (Figure 9). Overall, *Dm* and *Pa* inoculations showed stronger positive associations with plant growth parameters and photosynthetic pigments. *Pp* inoculation was more positively correlated with plant growth traits and several nutrient contents.

## 4. Discussion

### 4.1. DSE Tolerance to NaCl Stress In Vitro

As a primary growth metric, mycelial biomass production directly correlates with and effectively indicates fungal stress resistance capabilities in unfavorable conditions [43]. For the six DSE strains used in this experiment, heavy metal tolerance and drought tolerance have been reported in related studies [44,45], but reports on their salt tolerance are not known. All six DSE strains grew normally under NaCl stress, indicating their tolerance to NaCl stress. The study indicated that the total biomass of the DSEs decreased under NaCl stress conditions [46]. In our research, except for *Pp*, the biomass of the other five DSE strains decreased with increasing NaCl stress, whereas that of the *Pp* strain decreased and then increased. The stress resistance of living organisms can be increased by increasing their biomass production [47]. Under high NaCl stress, *Pp* demonstrated superior biomass accumulation compared to the other five strains, demonstrating its ability to enhance NaCl tolerance through biomass accumulation.

Fungal metabolism has been demonstrated to modify substrate pH through the release of organic acids and metabolic byproducts [16]. In this study, the pH of the culture solution of the *Dm* and *Aa* strains gradually increased with increasing NaCl concentration, which may be attributed to the specific secondary metabolites that are produced during the growth of the fungus [48].

Melanin can bind to the cell wall, enhancing its structural integrity and isolating it from harmful ions such as Na^+^ [49]. Additionally, melanin can act as an antioxidant to mitigate oxidative damage, which is regarded as an important feature of DSEs in stressful environments [50]. It had been reported melanin content may vary between fungi and may be affected by stress levels [51]. However, some studies have shown that melanin is not related to the tolerance of DSE strains in salt stress environments [36]. In our study, the *Ps* and *Dm* strains presented insignificant changes in melanin content across different NaCl concentrations, whereas the *Ex*, *Pp*, *Pa*, and *Aa* strains presented an increasing trend in melanin content with increasing NaCl stress, indicating that NaCl could induce melanin production in the *Ex*, *Pp*, *Pa*, and *Aa* strains. These findings suggest that melanin contributes to improving the NaCl tolerance of DSEs, but its effect is species-dependent.

MDA accumulation quantitatively reflects oxidative damage to cellular components [52], with increased levels indicating oxidative membrane damage and decreased levels demonstrating superior membrane stability [53]. In this study, the MDA content of the six DSE strains tended to increase with increasing NaCl concentration, suggesting that higher stress levels caused more severe membrane damage to the mycelium.

Under normal growth conditions, O^2-^ is balanced in organisms. However, abiotic stresses can adversely affect fungi, disrupting this balance and leading to the accumulation of reactive oxygen species (ROS), which can cause oxidative injuries in fungal cells [54]. SOD is a crucial protective enzyme that can protect cell membranes from various stress injuries [55]. In this study, the SOD activity in all six DSE strains first tended to increase, which was also observed for the soluble protein content. These findings suggest that the oxidative damage of DSE strains can be reduced by increasing their antioxidant enzyme activities. They may also regulate osmotic balance through the accumulation of soluble proteins to resist NaCl stress. This is consistent with the finding of Tan [19], which indicate that the increase in antioxidant enzyme activities and the accumulation of osmoregulatory substances under NaCl stress are important pathways for DSEs to resist NaCl stress. However, except for *Pp*, the SOD activity of the other five strains decreased when the NaCl concentration exceeded 0.8 M, suggesting that excessive NaCl compromises the ability of mycelia to efficiently remove excessive ROS from the organism, resulting in permanent damage to DSE strains [45]. In contrast, the SOD activity of *Pp* gradually increased with increasing NaCl stress, indicating that *Pp* maintained its ability to reduce oxidative damage. This phenomenon may be attributed to the increase in biomass accumulation under high NaCl stress, which collectively resulted in *Pp* showing less damage. Consequently, *Pp* presented lower levels of cellular damage and greater NaCl tolerance relative to the other strains under high NaCl stress.

### 4.2. Effects of DSE Inoculation on the Growth of Anemone tomentosa

Several studies have reported that DSEs can act as a plant growth promoter, positively affecting plant growth by influencing plant height and biomass. He et al. [44] found that inoculation with DSEs had a direct effect on the biomass and plant height of *Salvia miltiorrhiza* Bunge (Lamiaceae). With the exception of *Ps*, the other five DSE strains promoted the growth of *A. tomentosa*, suggesting that DSE inoculation can promote host plant growth to a certain extent, but this effect appears to be species-dependent [56,57].

The assessment of the fungal growth response revealed that the fungal growth response of *Dm*, *Pp*, and *Pa* was significantly greater than that of *Aa* and *Ex*. Endophytic fungi can form complex symbiotic relationships with plants, which may be neutral, reciprocal, or antagonistic due to environmental factors [58]. The fungal growth response is an index used to evaluate the symbiotic efficacy between root symbiotic fungi and plants, as well as the growth-promoting effect. A higher fungal growth response value indicates a stronger dependence between the host plant and mycorrhiza and a greater growth-promoting effect of the inoculated fungus. Therefore, compared with those of the *Aa* and *Ex* treatments, inoculation with *Dm*, *Pp*, and *Pa* had greater growth-promoting effects on the seedlings. In contrast, the fungal growth response of *Ps* was negative, indicating a weaker symbiotic ability with the host plant. These findings suggest that the beneficial effects of *Ps* inoculation on the host plant were insufficient to compensate for its nutrient depletion effects. Chu et al. [59] reported that the effects of DSEs on resistance to pine wilt disease in *Pinus sylvestris* L. (Pinaceae) were species-dependent, with the inoculation effect being positive, neutral, or negative depending on the fungal strain. Furthermore, the variation in the responses of the same host plant to different DSE species emphasizes the importance of selecting fungal species on the basis of their growth-promoting effects on host plants. This highlights the critical role of screening for fungal species with high compatibility with host plants in plant–fungus interactions to maximize the benefits of symbiotic associations.

Plants establish symbiotic relationships with microorganisms, and microorganisms with mycelia structures can extend into the soil to obtain nutrients for the host. Endophytic fungi, in particular, promote plant growth by transforming and solubilizing nutrients, including N, P, and K, making them more accessible for plant uptake [60,61,62,63]. In this study, all six inoculation treatments increased the root N content, whereas inoculation with *Pp* significantly increased the shoot N content and root P content. The ability of DSEs to promote N and P uptake in plants may be associated with the release of nutrients facilitated by various hydrolytic enzymes produced by these fungi [64]. Jumpponen and Trappe [9] reported that DSEs secrete a range of enzymes, including proteolytic enzymes, lipases, amylases, laccases, and polyphenol oxidases. These enzymes increase the release of mineral nutrients and help DSEs degrade various organic compounds, thereby increasing the accumulation of mineral nutrients in the host plant [58,65,66,67].

Inoculation with all six DSEs significantly increased the content of photosynthetic pigments in the leaves of *A. tomentosa*, suggesting that inoculation with DSEs can affect the content of chlorophyll in the host plant, which is similar to previous studies. Zhang et al. [60] reported that DSE inoculation enhanced chlorophyll content in *Lycium chinense* Mill. (Solanaceae). In summary, except for *Ps*, DSE inoculation generally had beneficial effects on the growth of *A. tomentosa*, although the extent of growth promotion varied among fungal species.

## 5. Conclusions

In vitro experiments revealed that all six DSE strains presented NaCl tolerance, among which the *Pp* strain presented greater NaCl tolerance than the other five strains. The results of the pot experiments revealed that all six DSE strains can successfully establish a symbiotic relationship with *A. tomentosa* and that *Dm*, *Pp*, *Pa*, *Aa*, and *Ex* promoted the growth of *A. tomentosa* seedlings, among which *Dm*, *Pp*, and *Pa* significantly promoted the growth of *A. tomentosa.* In conclusion, *Pp* establishes a mutually beneficial symbiotic relationship with *A. tomentosa*, influencing its nutrient uptake. Furthermore, *Pp* showed strong NaCl tolerance in the in vitro stress test. As a NaCl-tolerant strain with significant growth-promoting effects, *Pp* has the potential applications as a salt-tolerant microbial agent that can be used as a strain for subsequent NaCl stress pot experiments. This makes it particularly valuable for ecological restoration efforts in saline soil.

## Figures and Tables

**Table 1:** Two-way analysis of variance (ANOVA) for the effects of NaCl stress (NaCl) and dark septate endophyte (DSE) species on colony diameter (diameter), biomass, pH, soluble protein (SP) content, superoxide dismutase (SOD) activity, melanin content, malondialdehyde (MDA) content, and soluble sugar (SS) content of six DSE strains (*Didymella macrostoma*, *Paraboeremia selaginellae*, *Paraphoma pye*, *Paraphoma aquatica*, *Acrocalymma ampeli*, and *Exophiala xenobiotica*). *** indicates significance at *p* ≤ 0.001.

	DSE		NaCl		DSE × NaCl	
	*F*	*P*	*F*	*P*	*F*	*P*
Diameter	804.69	***	368.69	***	27.31	***
Biomass	46.15	***	67.81	***	8.48	***
pH	88.41	***	4.03	***	14.02	***
SP	483.57	***	102.87	***	40.89	***
SOD	465.11	***	34.53	***	52.56	***
Melanin	122.38	***	45.43	***	21.19	***
MDA	36.53	***	19.89	***	6.04	***
SS	98.50	***	68.28	***	32.41	***

## Data Availability

All data of this research are reported in the main text and Supplementary Materials; further inquiries can be directed to the corresponding author.

## Supplementary Materials

The following supporting information can be downloaded at: https://www.mdpi.com/article/10.3390/microorganisms13061303/s1. Figure S1: Morphology of fungal colonies and mycelium grown on potato dextrose agar (PDA) medium. (A–F) Colony morphology of *Didymella macrostoma* (*Dm*), *Paraboeremia selaginellae* (*Ps*), *Paraphoma pye* (*Pp*), *Paraphoma aquatica* (*Pa*), *Acrocalymma ampeli* (*Aa*), and *Exophiala xenobiotica* (*Ex*). (a–f) Mycelial morphology of *Dm*, *Ps*, *Pp*, *Pa*, *Aa*, and *Ex*. Scale bars = 50 μm. Figure S2: Phylogenetic tree based on 18S rDNA gene ITS sequence analysis of fungi isolated from roots of *Anemone tomentosa*. Sequences that were determined in the course of this study appear in bold. Fungus 1: *Didymella macrostoma*; Fungus 2: *Paraboeremia selaginellae*; Fungus 3: *Paraphoma pye*; Fungus 4: *Paraphoma aquatic*; Fungus 5: *Acrocalymma ampeli*; Fungus 6: *Exophiala xenobiotica*.

## References

[1] Li J.G., Pu L.J., Han M.F., Zhu M., Zhang R.S., Xiang Y.Z. (2014). Soil salinization research in China: Advances and prospects. J. Geogr. Sci..

[2] Santander C., Aroca R., Ruiz-Lozano J.M., Olave J., Cartes P., Borie F., Cornejo P. (2017). Arbuscular mycorrhiza effects on plant performance under osmotic stress. Mycorrhiza.

[3] Sanwal S.K., Kumar P., Kesh H., Gupta V.K., Kumar A., Kumar A., Meena B.L., Colla G., Cardarelli M., Kumar P. (2022). Salinity stress tolerance in potato cultivars: Evidence from physiological and biochemical traits. Plants.

[4] Turan M., Ekinci M., Kul R., Boynueyri F.G., Yildirim E. (2022). Mitigation of salinity stress in cucumber seedlings by exogenous hydrogen sulfide. J. Plant Res..

[5] Wang F.Y., Sun Y.H., Shi Z.Y. (2019). Arbuscular mycorrhiza enhances biomass production and salt tolerance of sweet sorghum. Microorganisms.

[6] Hill E.M., Robinson L.A., Abdul-Sada A., Vanbergen A.J., Hodge A., Hartley S.E. (2018). Arbuscular mycorrhizal fungi and plant chemical defence: Effects of colonisation on aboveground and belowground metabolomes. J. Chem. Ecol..

[7] Ruiz-Lozano J.M., Porcel R., Azcón C., Aroca R. (2012). Regulation by arbuscular mycorrhizae of the integrated physiological response to salinity in plants: New challenges in physiological and molecular studies. J. Exp. Bot..

[8] Liu H.G., Wang Y.J., Hart M., Chen H., Tang M. (2016). Arbuscular mycorrhizal symbiosis regulates hormone and osmotic equilibrium of *Lycium barbarum* L. under salt stress. Mycosphere.

[9] Jumpponen A., Trappe J.M. (1998). Dark septate endophytes: A review of facultative biotrophic root-colonizing fungi. New Phytol..

[10] Bilal S., Shahzad R., Imran M., Jan R., Kim K.M., Lee I.J. (2020). Synergistic association of endophytic fungi enhances *Glycine max* L. resilience to combined abiotic stresses: Heavy metals, high temperature and drought stress. Ind. Crops Prod..

[11] Hou L.F., He X.L., Li X., Wang S.J., Zhao L.L. (2019). Species composition and colonization of dark septate endophytes are affected by host plant species and soil depth in the Mu Us sandland, northwest China. Fungal Ecol..

[12] Lugo M.A., Reinhart K.O., Menoyo E., Crespo E.M., Urcelay C. (2015). Plant functional traits and phylogenetic relatedness explain variation in associations with root fungal endophytes in an extreme arid environment. Mycorrhiza.

[13] Mateu M.G., Baldwin A.H., Maul J.E., Yarwood S.A. (2020). Dark septate endophyte improves salt tolerance of native and invasive lineages of *Phragmites australis*. ISME J..

[14] Jumpponen A., Mattson K.G., Trappe J.M. (1998). Mycorrhizal functioning of *Phialocephala fortinii* with *Pinus contorta* on glacier forefront soil: Interactions with soil nitrogen and organic matter. Mycorrhiza.

[15] Santos M., Cesanelli I., Diánez F., Sánchez-Montesinos B., Moreno-Gavíra A. (2021). Advances in the role of dark septate endophytes in the plant resistance to abiotic and biotic stresses. J. Fungi.

[16] Xu M.H., Li X., Ye Q.N., Gong F., He X.L. (2024). Occurrence of dark septate endophytes in *Phragmites australis* in the Baiyang Lake and their resistance to Cd stress. Pedosphere.

[17] Wang D., Xie Y.L., Zhang W.Y., Yao L., He C., He X.L. (2024). Study on the biological characteristics of dark septate endophytes under drought and cadmium stress and their effects on regulating the stress resistance of *Astragalus membranaceus*. J. Fungi.

[18] Qu D.H., Wu F.L., Zhao X.H., Zhu D.Z., Gu L., Yang L.N., Zhao W.W., Sun Y.D., Yang J.J., Tian W. (2022). A bZIP transcription factor VabZIP12 from blueberry induced by dark septate endocyte improving the salt tolerance of transgenic *Arabidopsis*. Plant Sci..

[19] Tan J.Y., Yue Z.C., Li S.T., Pan Y.Y., Chu Z.Y., Ban Y.H., Xu Z.Y. (2024). Alleviation of salt stress and changes in glycyrrhizic acid accumulation by dark septate endophytes in *Glycyrrhiza glabra* grown under salt stress. J. Agric. Food Chem..

[20] Hou L.F., Li X., He X.L., Zuo Y.L., Zhang D.D., Zhao L.L. (2021). Effect of dark septate endophytes on plant performance of *Artemisia ordosica* and associated soil microbial functional group abundance under salt stress. Appl. Soil Ecol..

[21] Zhan F.D., Li B., Jiang M., Qin L., Wang J.X., He Y.M., Li Y. (2017). Effects of a root-colonized dark septate endophyte on the glutathione metabolism in maize plants under cadmium stress. J. Plant Interact..

[22] Zhu L.L., Li T., Wang C.J., Zhang X.R., Xu L.J., Xu R.B., Zhao Z.W. (2018). The effects of dark septate endophyte (DSE) inoculation on tomato seedlings under Zn and Cd stress. Environ. Sci. Pollut. Res..

[23] Valli P.P.S., Muthukumar T. (2018). Dark Septate root endophytic fungus *Nectria haematococca* improves tomato growth under water limiting conditions. Indian J. Microbiol..

[24] Li X., He X.L., Zhou Y., Hou Y.T., Zuo Y.L. (2019). Effects of dark septate endophytes on the performance of *Hedysarum scoparium* under water deficit stress. Front. Plant Sci..

[25] Sun Y.X., Li M.Q., Liu J.C. (2008). Haemolytic activities and adjuvant effect of *Anemone raddeana* saponins (ARS) on the immune responses to ovalbumin in mice. Int. Immunopharmacol..

[26] Hu H.B., Zheng X.D., Jian Y.F., Liu J.X., Zhu J.H. (2011). Constituents of the root of *Anemone tomentosa*. Arch. Pharmacal Res..

[27] Wang Y. (2012). Studies on the Saponin Constituents of *Anemone tomentosa*. Master’s Thesis.

[28] Chen X., Lu J.C., He W.F., Chi H.D., Yamashita K.I., Manabe M.B., Kodama H.Y. (2009). Antiperoxidation activity of triterpenoids from rhizome of *Anemone raddeana*. Fitoterapia.

[29] Zhang L.T., Zhang Y.W., Takaishi Y., Duan H.Q. (2008). Antitumor triterpene saponins from *Anemone flaccida*. Chin. Chem. Lett..

[30] Long J. (2014). The Studies on the Constituents of the Root of *Anemone tomentosa* and Its Secondary Metabolites Bioactivity of Endophytic Fungi. Master’s Thesis.

[31] Giannopolitis C.N., Ries S.K. (1977). Superoxide dismutases: II. purification and quantitative relationship with water-soluble protein in seedlings. Plant Physiol..

[32] Zhang C., Liu F., Kong W.W., He Y. (2015). Application of visible and near-infrared hyperspectral imaging to determine soluble protein content in oilseed rape leaves. Sensors.

[33] Wang Y.L., Gao S.S., He X.Y., Li Y., Li P.Y., Zhang Y., Chen W. (2019). Growth, secondary metabolites and enzyme activity responses of two edible fern species to drought stress and rehydration in northeast China. Agronomy.

[34] Wang Y.N., Jie W.G., Peng X.Y., Hua X.Y., Yan X.F., Zhou Z.Q., Lin J.X. (2019). Physiological adaptive strategies of oil seed crop *Ricinus communis* early seedlings (cotyledon vs. true leaf) under salt and alkali stresses: From the growth, photosynthesis and chlorophyll fluorescence. Front. Plant Sci..

[35] Wang Y.N., Lin J.X., Huang S.C., Zhang L., Zhao W.N., Yang C.X. (2019). Isobaric tags for relative and absolute quantification-based proteomic analysis of *Puccinellia tenuiflora* inoculated with arbuscular mycorrhizal fungi reveal stress response mechanisms in alkali-degraded soil. Land Degrad Dev..

[36] Gaber D.A., Berthelot C., Camehl I., Kovács G.M., Blaudez D., Franken P. (2020). Salt stress tolerance of dark septate endophytes is independent of melanin accumulation. Front. Microbiol.

[37] Zhang Z.L., Qu W.J. (2003). Laboratory Instruction of Plant Physiology.

[38] Phillips J.M., Hayman D.S. (1970). Improved procedures for clearing roots and staining parasitic and vesicular-arbuscular mycorrhizal fungi for rapid assessment of infection. Trans. Br. Mycol. Soc..

[39] Zhou Y., Zheng Y.Y., Li P.W., Xu L.J., Fu Q. (2024). Ectomycorrhizal fungi and dark septate endophyte inoculation improve growth and tolerance of *Pinus tabulaeformis* under cadmium stress. Pedosphere.

[40] Xu L.J., Li Y.H., Dai X.Y., Jin X.Y., Zhao Q.N., Tian B.Y., Zhou Y. (2025). Symbiotic fungal inoculation promotes the growth of *Pinus tabuliformis* seedlings in relation to the applied nitrogen form. BMC Plant Biol..

[41] Bao S.D. (2000). Agrochemical Analysis of Soil.

[42] Zhu S.P., Nong J.F., Luo G.T., Li Q.P., Wang F.S., Jiang D., Zhao X.C. (2021). Varied tolerance and different responses of five citrus rootstocks to acid stress by principle component analysis and orthogonal analysis. Sci. Hortic..

[43] Berthelot C., Leyval C., Foulon J., Chalot M., Blaudez D. (2016). Plant growth promotion, metabolite production and metal tolerance of dark septate endophytes isolated from metal-polluted poplar phytomanagement sites. FEMS Microbiol. Ecol..

[44] Lin Y.L., Wang Z.Z., Chen W.J., Liu Y.F., Li X., Tang H.L., He X.L. (2025). *Paraboremia selaginellae* enhances *Salvia miltiorrhiza* growth and cadmium tolerance via modulating root architecture and cadmium speciation in contaminated environments. Front. Plant Sci..

[45] He C., Wang W.Q., Hou J.L., Li X.N. (2021). Dark septate endophytes isolated from wild licorice roots grown in the desert regions of northwest China enhance the growth of host plants under water deficit stress. Front. Microbiol.

[46] Lalthazuali E., Marwein B., Sailo H., Lalbiaktluanga P.S., Vanlalmuana M.C.F., Ralte L. (2025). Enhancing rice growth in adverse conditions: The role of dark septate endophytes in salt and water scarcity tolerance. Mycologia.

[47] Ibrahim L., Proe M.F., Cameron A.D. (1998). Interactive effects of nitrogen and water availabilities on gas exchange and whole-plant carbon allocation in poplar. Tree Physiol..

[48] Zhang F., Zou Y.N., Wu Q.S., Kuca K. (2020). Arbuscular mycorrhizas modulate root polyamine metabolism to enhance drought tolerance of trifoliate orange. Environ. Exp. Bot..

[49] Kejžar A., Gobec S., Plemenitaš A., Lenassi M. (2013). Melanin is crucial for growth of the black yeast *Hortaea werneckii* in its natural hypersaline environment. Fungal Biol.

[50] Eisenman H.C., Casadevall A. (2012). Synthesis and assembly of fungal melanin. Appl. Microbiol. Biotechnol..

[51] Xie Y.L., He X.L., Wang D., Wang M.H., Li W.Y., Chen W.J., Li X.N., He C. (2024). Characterization of dark septate endophytes under drought and rehydration and their compensatory mechanisms in *Astragalus membranaceus*. Microorganisms.

[52] Wang W.B., Kim Y.H., Lee H.S., Kim K.Y., Deng X.P., Kwak S.S. (2009). Analysis of antioxidant enzyme activity during germination of alfalfa under salt and drought stresses. Plant Physiol. Biochem..

[53] Quiroga G., Erice G., Aroca R., Zamarreño A.M., García-Mina J.M., Ruiz-Lozano J.M. (2020). Radial water transport in arbuscular mycorrhizal maize plants under drought stress conditions is affected by indole-acetic acid (IAA) application. J. Plant Physiol..

[54] Deinlein U., Stephan A.B., Horie T., Luo W., Xu G.H., Schroeder J.I. (2014). Plant salt-tolerance mechanisms. Trends Plant Sci..

[55] Raja V., Majeed U., Kang H., Andrabi K.I., John R. (2017). Abiotic stress: Interplay between ROS, hormones and MAPKs. Environ. Exp. Bot..

[56] Wu L., Guo S. (2008). Interaction between an isolate of dark-septate fungi and its host plant *Saussurea involucrata*. Mycorrhiza.

[57] Gibert A., Tozer W., Westoby M. (2019). Plant performance response to eight different types of symbiosis. New Phytol..

[58] Surono, Narisawa K. (2017). The dark septate endophytic fungus *Phialocephala fortinii* is a potential decomposer of soil organic compounds and a promoter of *Asparagus officinalis* growth. Fungal Ecol..

[59] Chu H.L., Wang C.Y., Li Z.M., Wang H.H., Xiao Y.G., Chen J., Tang M. (2019). The dark septate endophytes and ectomycorrhizal fungi effect on *Pinus tabulaeformis* Carr. seedling growth and their potential effects to pine wilt disease resistance. Forests.

[60] Verma S.K., Sahu P.K., Kumar K., Pal G., Gond S.K., Kharwar R.N., White J.F. (2021). Endophyte roles in nutrient acquisition, root system architecture development and oxidative stress tolerance. J. Appl. Microbiol..

[61] White J.F., Kingsley K.L., Verma S.K., Kowalski K.P. (2018). Rhizophagy cycle: An oxidative process in plants for nutrient extraction from symbiotic microbes. Microorganisms.

[62] Soares M.A., Li H.Y., Kowalski K.P., Bergen M., Torres M.S., White J.F. (2016). Functional role of bacteria from invasive *Phragmites australis* in promotion of host growth. Microb. Ecol..

[63] White J.F., Kingsley K.L., Zhang Q.W., Verma R., Obi N., Dvinskikh S., Elmore M.T., Verma S.K., Gond S.K., Kowalski K.P. (2019). Review: Endophytic microbes and their potential applications in crop management. Pest Manag. Sci..

[64] Mandyam K., Jumpponen A. (2005). Seeking the elusive function of the root-colonising dark septate endophytic fungi. Stud. Mycol..

[65] Caldwell B.A., Jumpponen A., Trappe J.M. (2000). Utilization of major detrital substrates by dark-septate, root endophytes. Mycologia.

[66] Menkis A., Allmer J., Vasiliauskas R., Lygis V., Stenlid J., Finlay R. (2004). Ecology and molecular characterization of dark septate fungi from roots, living stems, coarse and fine woody debris. Mycol. Res..

[67] Mandyam K., Loughin T., Jumpponen A. (2010). Isolation and morphological and metabolic characterization of common endophytes in annually burned tallgrass prairie. Mycologia.

