# Supplementary material for: In Vitro Screening of NaCl-Tolerant Dark Septate Endophytes and Their Growth-Promoting Effects on Anemone tomentosa
Source: Microorganisms. 2025 Jun 4;13(6):1303. doi: 10.3390/microorganisms13061303 (PMC12194826; doi:10.3390/microorganisms13061303)
Supplement: Supplementary file 1 [file microorganisms-13-01303-s001.zip › microorganisms-3608772-supplementary.pdf]

In Vitro Screening of NaCl-Tolerant Dark Septate Endophytes and Their Growth-Promoting Effects on *Anemone tomentosa*

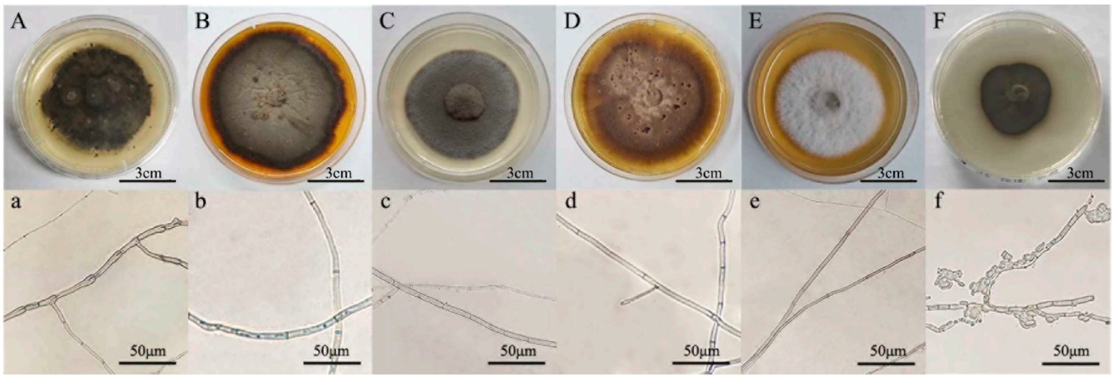

Figure S1: Morphology of fungal colonies and mycelium grown on potato dextrose agar (PDA) medium. (**A-F**) indicate colony morphology of *Didymella macrostoma* (*Dm*), *Paraboeremia selaginellae* (*Ps*), *Paraphoma pye* (*Pp*), *Paraphoma aquatica* (*Pa*), *Acrocalymma ampeli* (*Aa*), and *Exophiala xenobiotica* (*Ex*). (**a-f**) indicate mycelial morphology of *Dm*, *Ps*, *Pp*, *Pa*, *Aa*, and *Ex*. Scale bars = 50 μm.

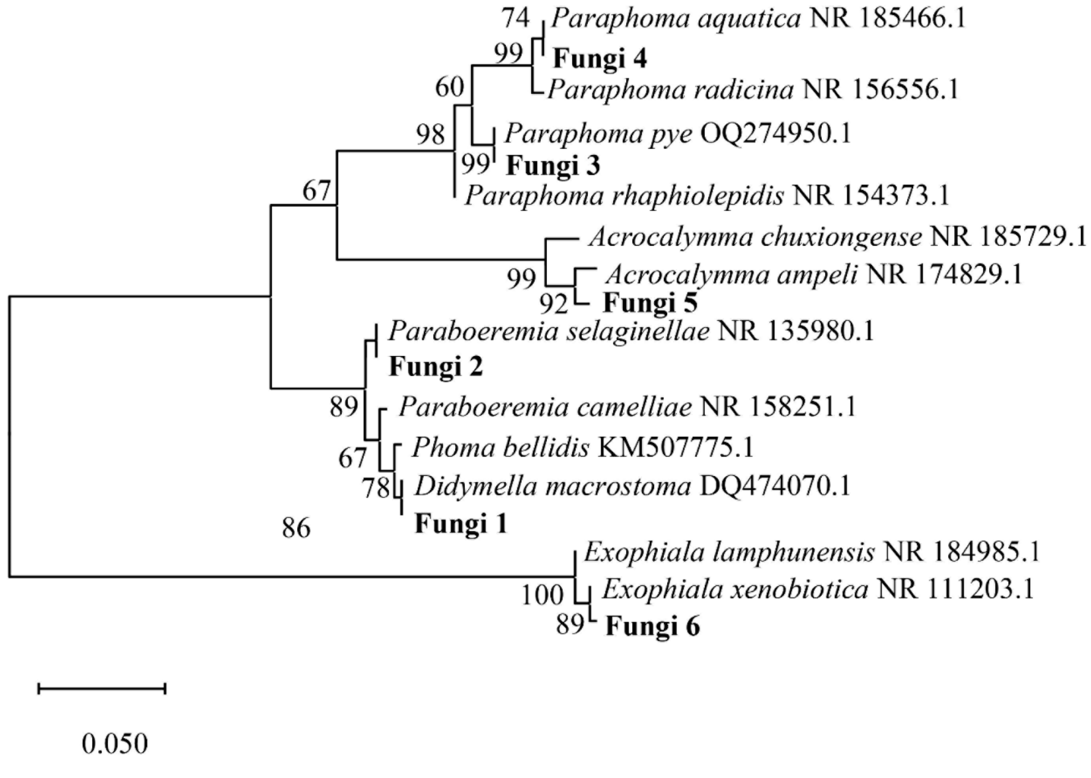

Figure S2: Phylogenetic tree based on 18S rDNA gene ITS sequence analysis of fungi isolated from roots of *Anemone tomentosa*. Sequences that were determined in the course of this study appear in bold. Fungi 1: *Didymella macrostoma*; Fungi 2: *Paraboeremia selaginellae*; Fungi 3: *Paraphoma pye*; Fungi 4: *Paraphoma aquatic*; Fungi 5: *Acrocalymma ampeli*; Fungi 6: *Exophiala xenobiotica*.
